# Functional connectivity analysis of fMRI data collected from human subjects with chronic tinnitus and varying levels of tinnitus-related distress

**DOI:** 10.1016/j.dib.2018.10.044

**Published:** 2018-10-19

**Authors:** Jeffrey Hullfish, Ian Abenes, Silvia Kovacs, Stefan Sunaert, Dirk De Ridder, Sven Vanneste

**Affiliations:** a School of Behavioral and Brain Sciences, The University of Texas at Dallas, Richardson, TX 75080, USA; b Translational MRI, Department of Imaging and Pathology & Medical Imaging Research Center, Department of Radiology, Catholic University of Leuven, Leuven 3000, Belgium; c Department of Surgical Sciences, Section of Neurosurgery, Dunedin School of Medicine, University of Otago, Dunedin 9054, New Zealand

## Abstract

The data presented here are functional connectivity analyses based on fMRI scans from a clinical sample of the chronic tinnitus population (*n* = 75). All data were obtained during an experiment in which subjects listened to auditory stimuli via headphones while undergoing fMRI scanning. The stimuli consisted of tones and bandpass noise presented at different frequencies. Stimulus frequency was the experimental factor, which was set (1) at each subject’s tinnitus percept frequency (TF) and (2) at an unrelated control frequency (CF) at least one octave away from the TF stimuli. All subjects were presented with stimuli at these two frequencies. We refer the reader to our original research article “Functional brain changes in auditory phantom perception evoked by different stimulus frequencies” (Hullfish et al., 2018) for further discussion. Here, we present data specifically from group-level analyses where the subjects were divided according to their level of tinnitus-related distress. The high-distress (HD) group comprised 43 subjects with Tinnitus Questionnaire (TQ) scores greater than or equal to 47, out of a possible 82 points. The low-distress (LD) group comprised the remaining 32 subjects with TQ score less than 47. The data presented include contrasts of functional connectivity elicited by TF and CF stimuli in each group as well as contrasts between the two groups.

**Specifications table**

**Table:** 

Subject area	Biology
More specific subject area	Neuroscience
Type of data	Matrices of correlation coefficients (Pearson’s r) and subtraction analyses (Δ Fisher’s Z), presented in figures.
How data was acquired	fMRI: Philips 3T MRI scanner
Data format	Analyzed
Experimental factors	*Auditory stimulus frequency.* There were two experimental conditions, such that auditory stimuli were presented (1) at each patient’s tinnitus percept frequency (TF) and (2) at a control frequency at least one octave away from the tinnitus frequency (CF).
Experimental features	Subjects were scanned using a block-design fMRI paradigm while listening to auditory stimuli presented via headphones.
Data source location	1. University Hospital of Antwerp (Belgium) 2. Catholic University of Leuven (Belgium)
Data accessibility	Data are with this article
Related research article	J.A. Hullfish, I. Abenes, S. Kovacs, S. Sunaert, D. De Ridder, S. Vanneste, Functional brain changes in auditory phantom perception evoked by different stimulus frequencies, Neurosci. Lett. 683 (2018) 160–167. doi:10.1016/J.NEULET.2018.07.043. [1]

**Value of the data**

• The data represent a large, clinical sample of the human chronic tinnitus population (*n* = 75).
• The data offer a basis for further investigation of heterogeneity in tinnitus, especially with regards to tinnitus-related distress.
• The data offer a means to explore the different responses of tinnitus brains to auditory stimuli at the network level.

## 1 Data

The data presented here come from a functional connectivity analysis of fMRI data collected from a large sample (*n* = 75) of the chronic tinnitus population. The purpose of the analysis was to examine changes in functional connectivity elicited by different auditory stimuli in subjects with varying levels of tinnitus-related distress. Tinnitus-related distress, measured using the Tinnitus Questionnaire (TQ), was included as a covariate in the analysis to emphasize the group-level differences, i.e. between the high- and low-distress subgroups of our larger sample. These data are presented here as figures, which display partial correlation coefficients that reached significance at the .05 level, including the FDR correction for multiple comparisons.

## 2 Experimental design, materials, and methods

For a full description of the experimental design, materials, and methods, refer to the related research article [1]. The following only represents information unique to these specific analyses.

### 2.1 Subjects

The subject group consisted of humans with clinically relevant tinnitus (*n* = 75), defined as being severe enough for the patient to voluntarily seek out treatment [2]. Subjects reported tinnitus-related distress using the Tinnitus Questionnaire (TQ), which is scored from 0 to 82 with higher scores corresponding to greater levels of distress [3]; the mean TQ score was 49.3 ± 16.4 (SD). We categorized distress based on patients’ TQ scores: slight (0–30 points; grade 1), moderate (31–46; grade 2), severe (47–59; grade 3), and very severe (60–84; grade 4) distress [4]. Patients with grade 1–2 tinnitus were our low distress (LD, *n* = 32) group and patients with grade 3–4 tinnitus were our high distress (HD, *n* = 43) group.

### 2.2 Analysis

**Fig. 1:**
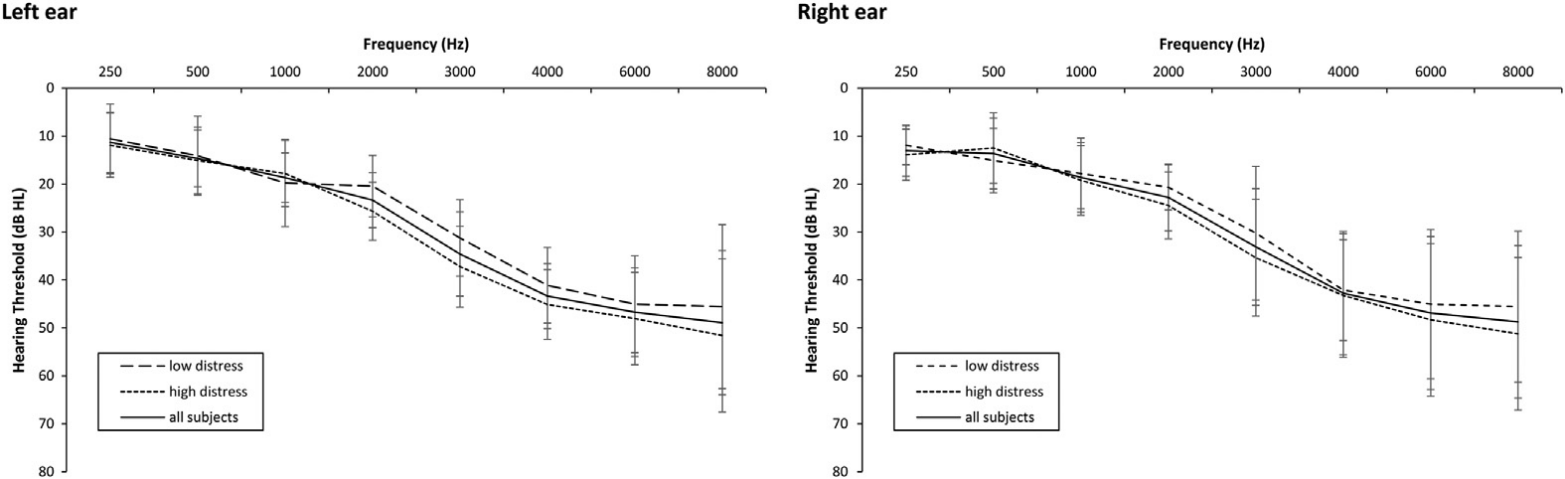
*Pure-tone audiograms.* Mean pure-tone hearing thresholds in dB HL at frequencies 250–8000 Hz (=0.250–8 kHz). The solid line indicates the mean thresholds for all subjects (*n* = 75), while the dashed lines indicate the LD and HD subgroups (*n* = 32 and 43, respectively). Error bars indicate the standard error at each frequency. MANOVA shows no significant differences in hearing thresholds between the groups.

Fig. 1 shows the average pure-tone hearing thresholds for the left and right ear for all tinnitus subjects. A MANOVA with hearing thresholds for all frequencies (dB HL: 0.25–8 kHz) as dependent variables and tinnitus group (LD vs. HD) as the independent variable showed no significant effect between the groups for all frequencies.

We analyzed functional connectivity between 19 regions of interest using partial correlations (Matlab partialcorr(): https://www.mathworks.com/help/stats/partialcorr.html). We determined partial correlations separately for HD and LD subjects, controlling for TQ within each group, in response to both TF and CF stimulation. By using TQ as a covariate, we center the effect of TQ at each group’s mean level. This better reflects the group-level differences in tinnitus-related distress, since our “low-distress” subgroup has a maximum TQ score of 46 (out of 82) and our “high-distress” subgroup has a minimum TQ score of 47. We checked all results for significance at the .05 level, including the FDR correction for multiple comparisons, using the method described in [5] (Matlab mafdr(): https://www.mathworks.com/help/bioinfo/ref/mafdr.html). We treated those partial correlations that survived FDR correction as functional connections, which we then visualized using heat-maps (Matlab HeatMap(): https://www.mathworks.com/help/bioinfo/ref/heatmap.html).

**Fig. 2:**
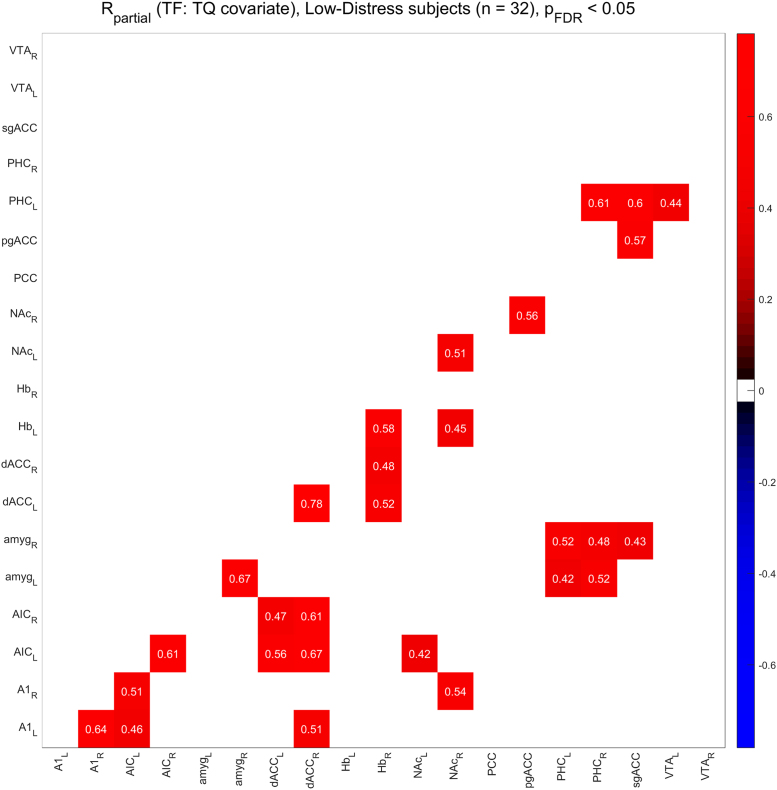
*Functional connectivity, LD subjects during TF stimulation.* Colored squares indicate partial correlations (i.e. functional connections) that reach significance at the .05 level, including the FDR correction for multiple comparisons. Connections were analyzed between 19 regions of interest, which are labeled along the axes of the table.

**Fig. 3:**
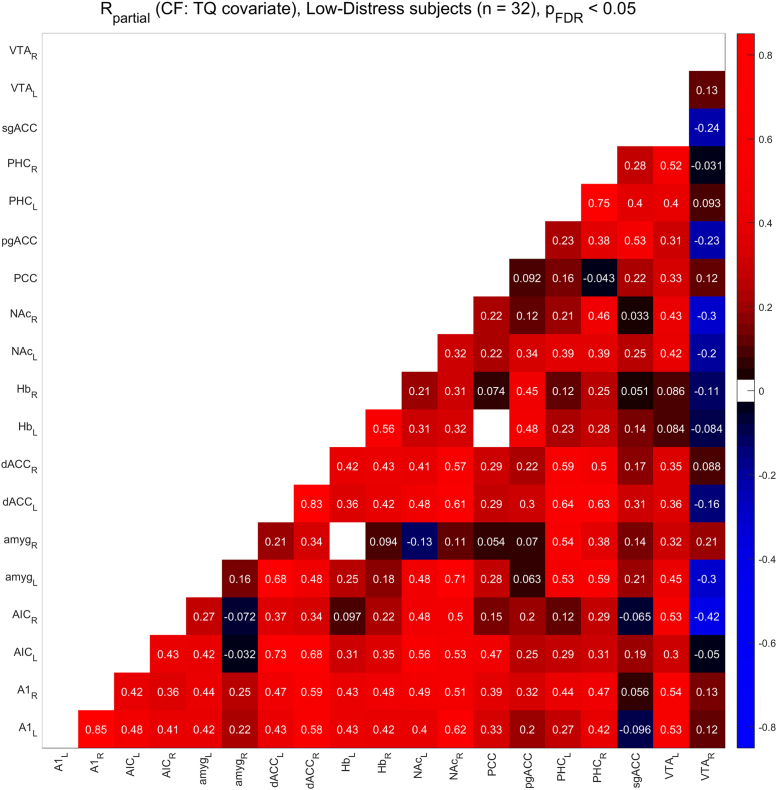
*Functional connectivity, LD subjects during CF stimulation.* Colored squares indicate partial correlations (i.e. functional connections) that reach significance at the .05 level, including the FDR correction for multiple comparisons. Connections were analyzed between 19 regions of interest, which are labeled along the axes of the table.

**Fig. 4:**
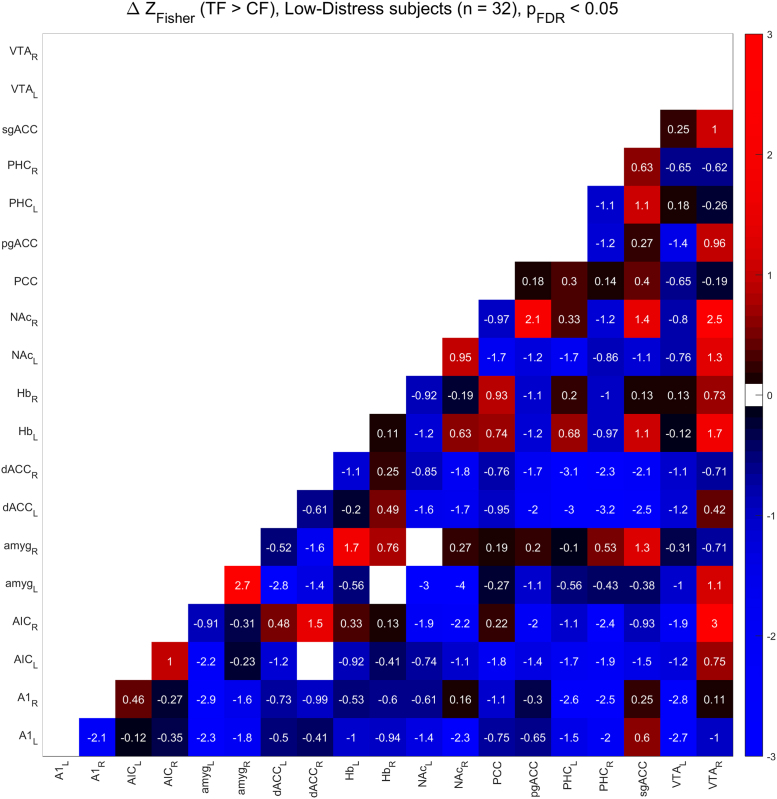
*Subtraction analysis, TF > CF, LD subjects.* Colored squares indicate changes in functional connectivity (i.e. differences in Fisher’s Z-transformed partial correlation coefficients) that reach significance at the .05 level, including the FDR correction for multiple comparisons. Differences were analyzed between 19 regions of interest, which are labeled along the axes of the table.

**Fig. 5:**
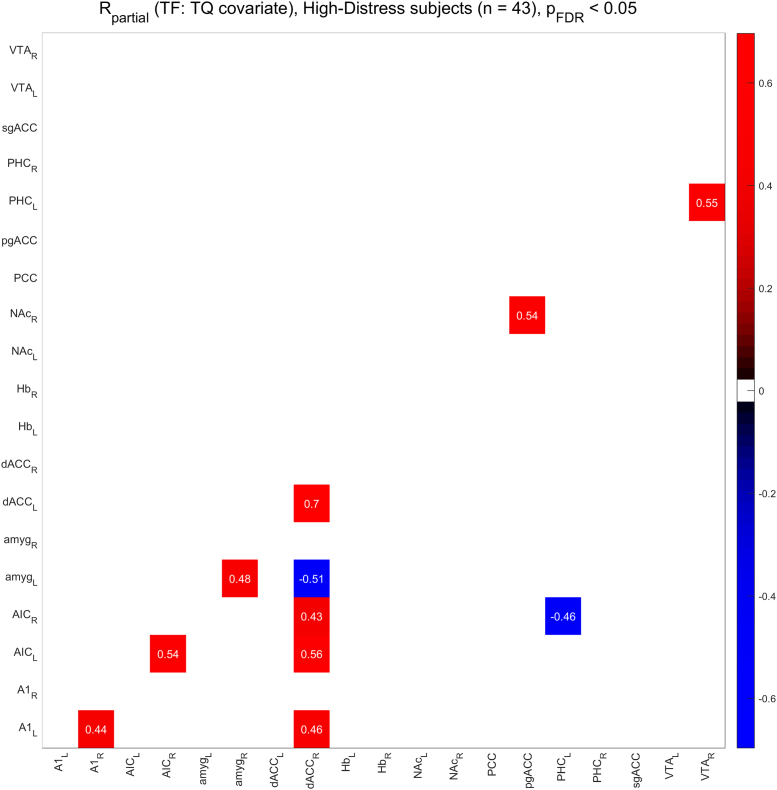
*Functional connectivity, HD subjects during TF stimulation.* Colored squares indicate partial correlations (i.e. functional connections) that reach significance at the .05 level, including the FDR correction for multiple comparisons. Connections were analyzed between 19 regions of interest, which are labeled along the axes of the table.

**Fig. 6:**
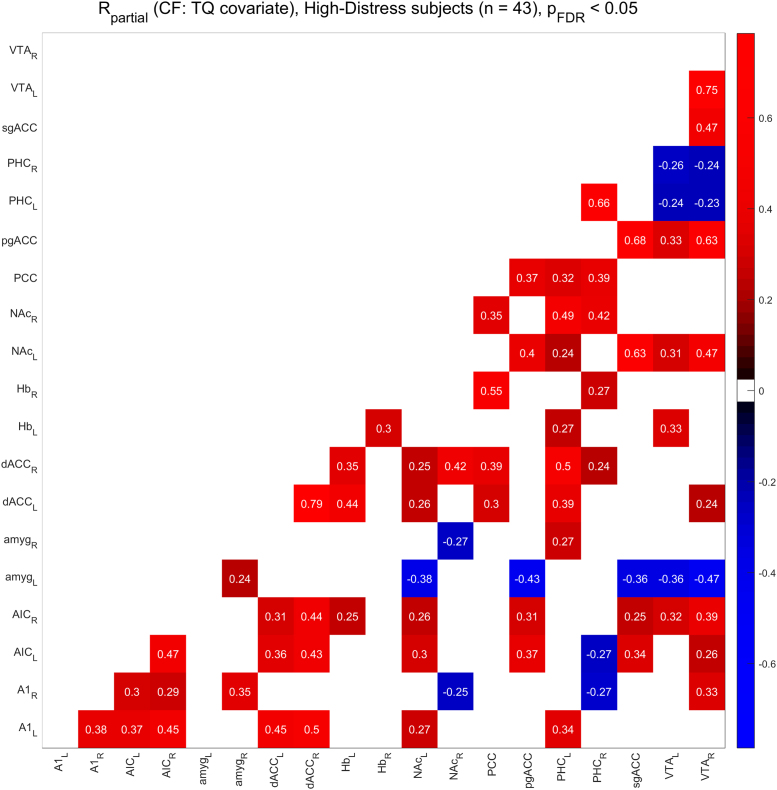
*Functional connectivity, HD subjects during CF stimulation.* Colored squares indicate partial correlations (i.e. functional connections) that reach significance at the .05 level, including the FDR correction for multiple comparisons. Connections were analyzed between 19 regions of interest, which are labeled along the axes of the table.

**Fig. 7:**
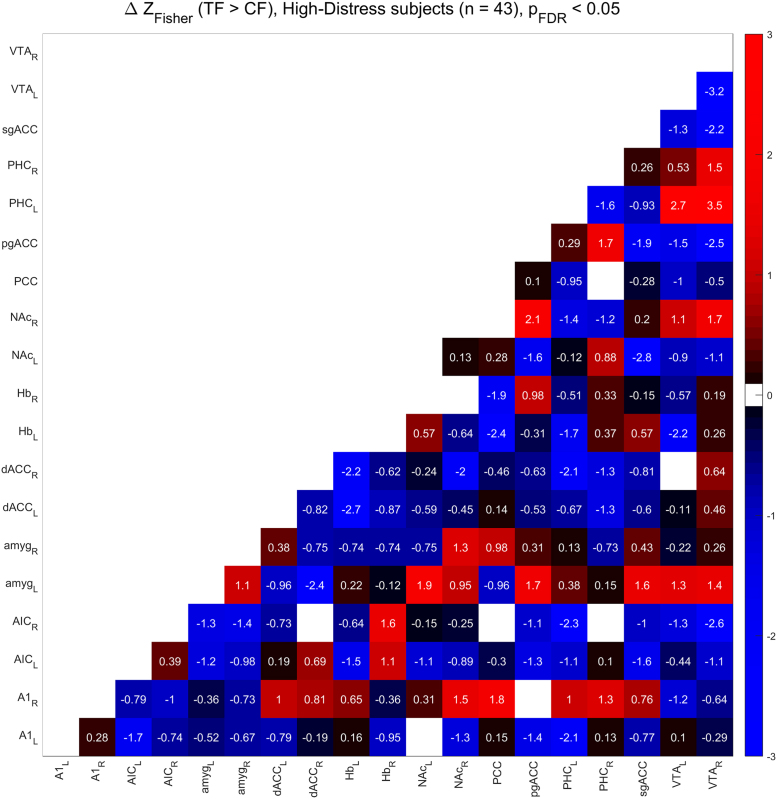
*Subtraction analysis, TF > CF, HD subjects.* Colored squares indicate changes in functional connectivity (i.e. differences in Fisher’s Z-transformed partial correlation coefficients) that reach significance at the .05 level, including the FDR correction for multiple comparisons. Differences were analyzed between 19 regions of interest, which are labeled along the axes of the table.

**Fig. 8:**
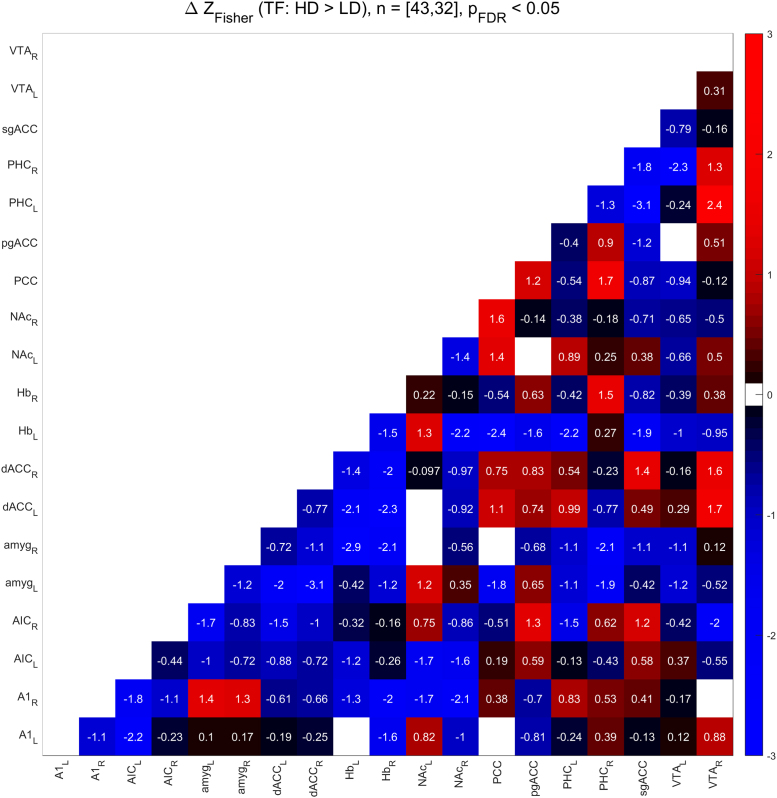
*Subtraction analysis, HD > LD, TF stimulation.* Colored squares indicate changes in functional connectivity (i.e. differences in Fisher’s Z-transformed partial correlation coefficients) that reach significance at the .05 level, including the FDR correction for multiple comparisons. Differences were analyzed between 19 regions of interest, which are labeled along the axes of the table.

**Fig. 9:**
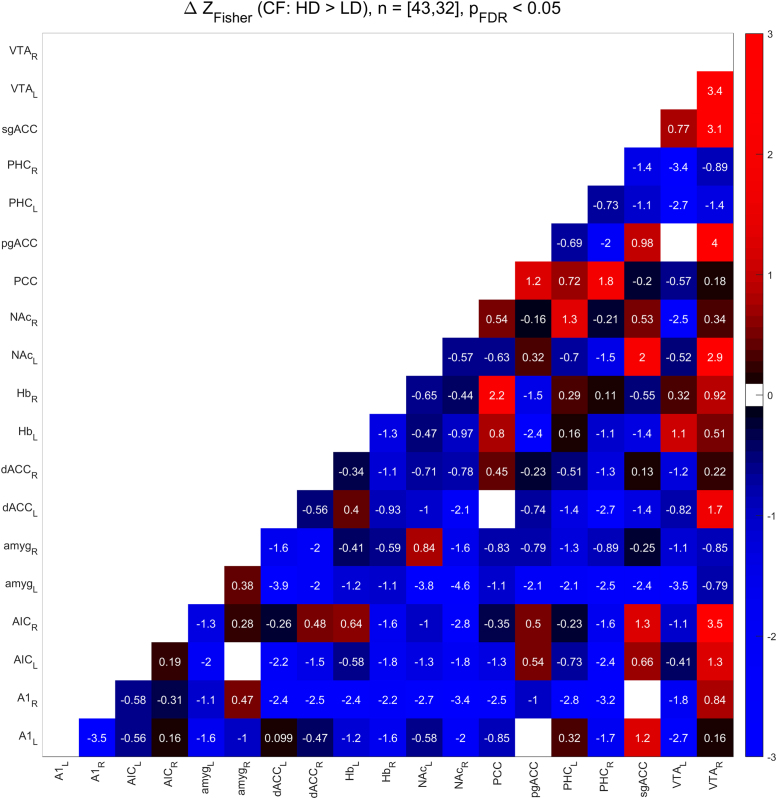
*Subtraction analysis, HD > LD, CF stimulation.* Colored squares indicate changes in functional connectivity (i.e. differences in Fisher’s Z-transformed partial correlation coefficients) that reach significance at the .05 level, including the FDR correction for multiple comparisons. Differences were analyzed between 19 regions of interest, which are labeled along the axes of the table.

Fig. 2, Fig. 3 show the functional connectivity in the LD subjects while Fig. 5, Fig. 6 show the same in the HD subjects. Furthermore, we compared partial correlations between groups by performing Fisher’s Z transformation on the results from each group, subtracting the two groups to be compared (e.g. TF vs. CF, HD vs. LD, etc.), and checking the differences for significance at the FDR-corrected .05 level [5]. Fig. 4 shows the difference between TF- and CF-evoked connectivity in the LD subjects, Fig. 7 shows the same in the HD subjects, Fig. 8 shows the difference between HD and LD subjects during TF, and Fig. 9shows the same during CF.
