# Supplementary material for: Functional connectivity analysis of fMRI data collected from human subjects with chronic tinnitus and varying levels of tinnitus-related distress
Source: Data Brief. 2018 Oct 19;21:779–89. doi: 10.1016/j.dib.2018.10.044 (PMC6216076; doi:10.1016/j.dib.2018.10.044)
Supplement: Supplementary file 1 — Transparency document [file mmc1.docx]

The authors have no conflict of interest
